# Comparison of Coagulation-Integrated Sand Filtration and Ultrafiltration for Seawater Reverse Osmosis Pretreatment

**DOI:** 10.3390/membranes14060125

**Published:** 2024-05-29

**Authors:** Qingao Li, Lixin Xie, Shichang Xu, Wen Zhang

**Affiliations:** Tianjin Key Laboratory of Membrane Science and Desalination Technology, State Key Laboratory of Chemical Engineering, School of Chemical Engineering and Technology, Tianjin University, Tianjin 300350, China; lqa@tju.edu.cn (Q.L.); xu_sc1@tju.edu.cn (S.X.)

**Keywords:** dissolved organic matter (DOM), coagulation, sand filtration (SF), ultrafiltration (UF), integrated pretreatment, reverse osmosis (RO), organic fouling

## Abstract

The removal of dissolved organic matter (DOM) from seawater before the reverse osmosis (RO) processes is crucial for alleviating organic fouling of RO membranes. However, research is still insufficiently developed in the comparison of the effectiveness of integrating coagulation with ultrafiltration (UF) or sand filtration (SF) in the pretreatment stage of seawater reverse osmosis (SWRO) for the removal of DOM. In this study, we investigated the effect of pretreatment technologies on RO fouling caused by DOM in seawater, including the integration of coagulation and sand filtration (C-S pretreatment) and the integration of coagulation and ultrafiltration (C-U pretreatment). Both integrated pretreatments achieved comparable DOM removal rates (70.2% for C-U and 69.6% for C-S), and C-S exhibited enhanced removal of UV-absorbing compounds. Although C-U was more proficient in reducing the silt density index (below 2) compared to C-S (above 3) and improved the elimination of humic acid-like organics, it left a higher proportion of tyrosine-protein-like organics, soluble microbial by-product-like organics, and finer organics in the effluent, leading to the formation of a dense cake layer on RO membrane and a higher flux decline. Therefore, suitable technologies should be selected according to specific water conditions to efficiently mitigate RO membrane fouling.

## 1. Introduction

Reverse osmosis (RO) has become the dominant technology in supplying freshwater from unlimited seawater [1,2]. However, organic fouling caused by dissolved organic matter (DOM) is still a significant technical obstacle in seawater RO desalination [3]. DOM is a complex and heterogeneous mixture consisting of various active organic species, including polysaccharides, proteins, and humic substances [3]. DOM plays a dominant role in the formation of fouling layers on RO membrane surfaces [4]. Consequently, the effective pretreatment of seawater to remove these potential contaminants is essential for the long-term stable operation of seawater reverse osmosis (SWRO) desalination [5,6].

Traditional RO pretreatment technologies, such as coagulation and granular media filtration, are widely employed in desalination processes. A paramount benefit of conventional pretreatment technologies lies in their longstanding implementation, which has proven their efficacy and rendered them a familiar technology within the field [7]. Nonetheless, these processes are marked by certain drawbacks, chief among them being a heightened sensitivity to fluctuations in source water properties. This variability necessitates the adjustment of treatment chemical doses [8]. Up-to-date low-pressure membrane technology, such as ultrafiltration (UF), is employed as the pretreatment technology to mitigate RO fouling [9]. It offers the advantages of little or no requirement for chemicals, reduced sludge production and a small plant footprint [10,11]. Notably, neither traditional pretreatments nor membrane technologies guarantee the comprehensive removal of DOM from the feedwater of RO systems [12,13,14]. Lin et al. [15] showed that aluminum-based coagulants remove more than 60% of fulvic acid-like organics in HA/AOM mixtures while exhibiting minimal removal of aromatic protein-like substances. Guo et al. [16] conducted a study on the performance of continuous sand filtration (SF) systems in treating DOM, and the results showed that the removal rate was only 11.1%. Liu et al. [17] observed that UF demonstrates limited efficacy in removing DOM, particularly for low molecular weight, hydrophilic fractions. Chua et al. [18] employed polyether sulfone (PES) hollow fiber UF membrane with a nominal pore size of 0.01 μm as pretreatment for SWRO membranes, observing remarkable removal of colloidal silica and a coliform group of bacteria, while only removing a small percentage of total organic carbon (TOC).

Due to the unsatisfactory performance of single pretreatment technology, integrated pretreatment technologies have been developed to achieve better performance in DOM removal. For example, coagulation integrated with UF can reduce the silt density index (SDI) of seawater to 0.8 [19]. Moreover, coagulation combined with dual-media filtration (DMF) is also more effective at removing dissolved organic carbon (DOC) than microfiltration [20]. Guastalli et al. [21] compared the effectiveness of DMF and UF membranes as pretreatment methods for seawater desalination systems in removing particles and DOM. Both techniques demonstrated efficacy in eliminating particulate and microbial contaminants, consistently maintaining water turbidity below 0.1 NTU and SDI under 2. However, the efficiency of DOM removal was found to be low for both pretreatments, with DMF achieving a 2% removal rate, and UF showing a slightly higher rate at 9%. The membrane-based UF and SF technologies are widely used pretreatment technologies. However, comparative studies on the effectiveness of integrating coagulation with either UF or SF to remove DOM in SWRO pretreatments remain insufficient.

**Figure 1:**
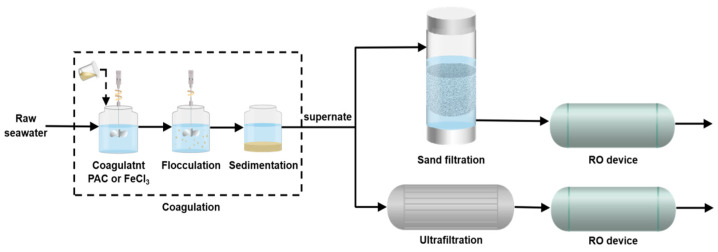
Schematic diagram of integrated seawater pretreatment processes.

This study aimed to compare the effectiveness of two integrated pretreatment technologies, the coagulation-sand filtration (C-S pretreatment) and coagulation-ultrafiltration (C-U pretreatment) methods, in removing DOM from seawater and reducing fouling in RO membranes (Figure 1). The effectiveness of different pretreatment technologies in DOM removal was assessed using comprehensive water quality indices such as SDI, turbidity, DOC, and UV_254_. Additionally, techniques like three-dimensional excitation-emission matrix (EEM) spectral analysis and hydrophobic/hydrophilic proportion were also utilized to further reveal the changes in DOM components and properties in the effluent from various pretreatment technologies. This study contributes to the optimization and enhancement of the pretreatment stage in the SWRO desalination process, and provides guidance for designing more-efficient and sustainable pretreatment schemes.

## 2. Materials and Methods

### 2.1. Materials

To simulate seawater, 32 g·L^−1^ NaCl (AR, Rionlon, Tianjin, China) was added to deionized water, the pH was adjusted to 8 ± 0.3 with NaOH (GR, Kermel, Tianjin, China), and the turbidity was adjusted to 15 ± 0.5 NTU using a suspension of kaolin clay (AR, Heowns, Tianjin, China). Humic acid (HA, M06273, Meryer, Shanghai, China), sodium alginate (SA, M76392, Meryer, Shanghai, China), and bovine serum albumin (BSA, FA016, Genview, Beijing, China) were used to simulate the humic substances, carbohydrates, and proteins commonly present in seawater. Each component was utilized at a concentration of 5 mg·L^−1^.

### 2.2. Pretreatment Experiments

Polyaluminum chloride (PAC, AR, Damao, China) and ferric chloride (FeCl_3_, AR, Aladdin, Shanghai, China) were chosen as coagulants. The doses employed were 5–50 mg·L^−1^ for PAC and 5–15 mg·L^−1^ for FeCl_3_. Coagulation tests were performed using a magnetic stirrer (ZR4-6, Zhongurn, Shenzhen, China) according to the following procedure. The mixture was stirred at 250 rpm for 30 s. After the addition of coagulants, it was rapidly stirred at 200 rpm for 90 s, followed by slow stirring at 40 rpm for 10 min. After a 30 min settling period, the supernatant was collected for analysis.

The SF device had an inner diameter of 30 mm and height of 1000 mm. The flow rate during filtration was controlled by a flow meter. The column was packed with homogeneous quartz sand, with a particle size in the range of 0.4–0.6 mm (0.45 mm average). The packing height was 550 mm.

All UF experiments were conducted in dead-end filtration mode using a PES hollow-fiber UF membrane (UIE230K, Motech, Tianjin, China) with a molecular weight cutoff of 35 kDa. The membrane, with an area of 0.2 m^2^, exhibited an average contact angle of 42.5°. Prior to the experiments, the UF membranes were soaked in deionized water for 12 h and then flushed with 250 mL of deionized water to remove the protective layer from the membrane surface and achieve a stable membrane flux. The UF unit was operated under constant flow conditions, with an influent flow rate of 50 L·m^−2^·h^−1^.

All experiments were repeated three times to ensure the reliability and accuracy of the experimental data.

### 2.3. RO Experiments

The RO membrane was a flat-sheet polyamide membrane purchased from Dow Chemical Company, with an effective area of 31.16 cm^2^. An RO device equipped with a cross-flow membrane cell was used to evaluate the performance of the RO membrane by monitoring the permeate flux and salt rejection rate under an operating pressure of 5.5 MPa. The system was operated at a cross-flow velocity of 1.5 L·min^−1^ for 30 min, and the water flux (*J*) was recorded every 10 min. The average water flux within 30 min was considered the initial water flux (*J*_0_), and the salt rejection rate was also recorded. After draining the feed tank, effluent from either the C-S pretreatment or C-U pretreatment was added to the feed tank to serve as the feed water for the RO system. The water flux *(J*) and salt rejection rate were recorded every 60 min. Normalized flux (*J*/*J*_0_) was employed to evaluate the extent of fouling on the RO membrane. All experiments were repeated three times.

### 2.4. Analytical Methods

Before analysis, the samples were filtered through a 0.45 μm membrane filter. The aromatic content of DOM in water samples was measured in terms of UV absorbance at 254 nm (UV_254_) with an ultraviolet visible spectrophotometer (TU-1900, Pgengal, Tianjin, China) [4]. The DOC content was determined using a total organic carbon analyzer (TOC-LCPN, Shimadzu, Kyoto, Japan). The specific UV absorbance (SUVA) was calculated using the ratio of DOC concentration and UV_254_, representing an index of DOM aromaticity [22]. The solution pH was obtained using a pH meter (FE28-CN, Zurich, Mettler Toledo, Switzerland). The zeta potential and particle size distribution of the solution were analyzed with a Nano ZS nanoparticle size and zeta potential analyzer (Zetasizer Nano ZS90, Malvern, Shanghai, China). Turbidity was determined using a turbidity meter (2100 N, Hach, CO, USA), and the solution conductivity was measured with a conductivity meter (DDS-11A, Leici, Shanghai, China). Inductively coupled plasma-mass spectrometry (ICP-MS) (5110(OES), Agilent, Santa Clara, CA, USA) was employed to determine the concentration of iron in the water samples. Approximately 5 mL of sample was required for the analysis of UV_254_, zeta potential, and particle size distribution. A volume of about 10 mL per test was necessary for measuring DOC and turbidity. Meanwhile, the determination of iron content required around 15 mL of sample.

DOM was fractionated into a hydrophobic (HPO) fraction, transphilic (TPI) fraction, and hydrophilic (HPI) fraction using XAD-4 and DAX-8 resins (Sigma-Aldrich, Wilmington, DE, USA). The HPO and TPI fractions was strongly absorbed by DAX-8 resin and XAD-4 resin, respectively, leaving only the HPI fraction left. For the XAD-4/DAX-8 resin analysis, the sample volume (V) was 150 mL, the column volume (V_0_) was 20 mL, and the titration rate (R_t_) was set as 40 mL·h^−1^ (2 times V_0_ per hour). Specifically, the pH of the sample was adjusted to 2 by adding HCl, and then the DAX-8 resin was used to adsorb the HPO fraction. After DAX-8 adsorption, one part of the sample was taken for DOC detection, and then the remaining sample was adsorbed using XAD-4 resin. Finally, the HPI (after XAD-4 adsorption) and HPO (after DAX-8 adsorption) content of the DOM was calculated by measuring the DOC values of the original acidified samples [23]. The SDI was calculated in accordance with the American Society for Testing and Materials (ASTM) standards [24].

A three-dimensional EEM was employed to characterize the fluorescent components in the raw and treated water. EEM spectra were generated using a fluorescence spectrophotometer (F-4600, Hitachi, Tokyo, Japan) with the emission (Em) wavelengths of 200–550 nm in 5 nm increments, and the excitation (Ex) wavelengths of 200–450 nm in 5 increments. The fluorescence regional integration (FRI) method was applied to analyze EEM spectra. The ratio of the fluorescence response per unit area of each region (*Φ_i,n_*) to the total fluorescence response per unit area (*Φ_T,n_*) was defined as the fluorescence response percentage (*P_i,n_* = *Φ_i,n__/_Φ_T,n_* × 100%), reflecting the relative abundance of specific fluorescent substances [25]. Each EEM analysis required approximately 5 mL of water sample.

A scanning electron microscope (SEM) equipped with an energy dispersive X-ray spectrometer (EDX) (Regulus 8100, Hitachi, Tokyo, Japan) was utilized to observe the surface microstructures of the RO membrane and quartz sand. The examination was conducted at an accelerating voltage of 3 kV, employing magnifications of 20 k times for the RO membrane and 40 k times for the quartz sand. The surface morphology and roughness of the membrane were obtained with an atomic force microscope (AFM) (Bruker Dimension Icon, Karlsruhe, Germany). The AFM was operated with an RPESPA-75 cantilever. To improve the accuracy of the results, the arithmetic average roughness (Ra) of RO membranes was measured at three points in each sample. Changes in the specific surface area of the quartz sand filter media before and after operation were measured using an automatic surface area analyzer (AutoSorb-iQ2-MP, Quanta, Fremont, CA, USA). The water contact angle of the membrane surface was measured through a contact angle goniometer (OCA20, Dataphysics, Stuttgart, Germany). A volume of 2 mL of DI water was dropped onto the dried membrane surface. Measurements were repeated 5 times to calculate the average contact angle. The zeta potential of the membrane surface was determined using a solid surface zeta potential analyzer (SurPASS 3, Anton Paar, Graz, Austria). In the measurement process, the zeta potential of the RO membranes was determined in a 1 mM KCl aqueous solution at pH 5.5, with a gap height maintained at 100 ± 5 µm. Measurements were taken in triplicate, and the average zeta potential value was reported.

## 3. Results

### 3.1. Optimization of Coagulants

**Figure 2:**
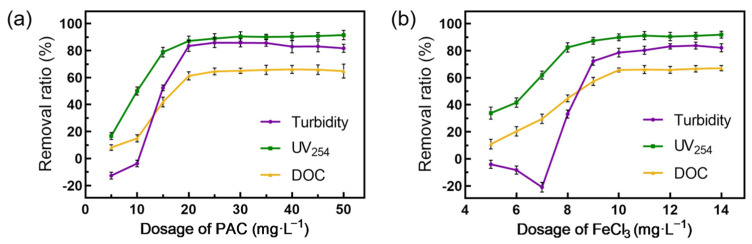
Removal ratio of turbidity, UV_254_ and DOC of different coagulants in the coagulation treatment of the raw seawater. (**a**) PAC, (**b**) FeCl_3_.

The coagulants PAC and FeCl_3_ are used for seawater treatment. In Figure 2, the removal performance of DOC and UV_254_ by two types of coagulants show a similar trend. That is, as the doses of coagulants increases, the removal rates also rise. However, the rate of increase slows down at higher doses. Both coagulants demonstrated significantly greater removal rates for UV_254_ than DOC, indicating superior effectiveness in removing aromatic compounds with unsaturated bonds. Compared with those of PAC, the removal rates of FeCl_3_ were slightly greater for UV_254_ and DOC, which is consistent with previous research results [26]. Additionally, both coagulants achieved turbidity removal rates exceeding 80%, indicating effective clarification performance. When the dose of FeCl_3_ was below 7 mg·L^−1^, it only resulted in the formation of non-settling small flocs, leading to an increase in the turbidity of the supernatant.

**Figure 3:**
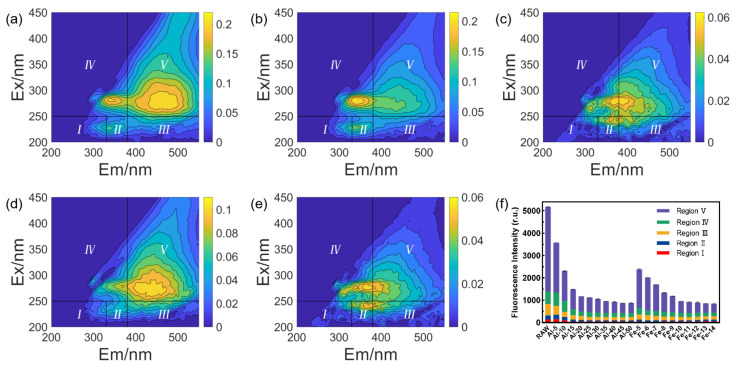
EEM spectra and FRI analysis of organics in effluent before or after coagulation. (**a**) Raw water, (**b**) PAC at a concentration of 5 mg·L^−1^, (**c**) PAC at a concentration of 30 mg·L^−1^, (**d**) FeCl_3_ at a concentration of 5 mg·L^−1^, (**e**) FeCl_3_ at a concentration of 10 mg·L^−1^, (**f**) fluorescence intensity of effluent after being pretreated by PAC or FeCl_3_ (Regions I, II, III, IV, and V were noted in the EEM spectras, corresponding to tyrosine protein-like organics, tryptophan protein-like organics, fulvic acid-like organics, soluble microbial by-product-like (SMP-like) organics, and humic acid-like organics, respectively).

The EEM spectra in Figure 3 were analyzed to provide a comprehensive characterization of the organic substances in the water samples before and after coagulation pretreatment. Five regions, namely, region I, region II, region III, region IV and region V, were observed in the EEM spectra, relating to tyrosine protein-like organics, tryptophan protein-like organics, fulvic acid-like organics, soluble microbial by-product-like (SMP-like) organics and humic acid-like organics, respectively [25]. In Figure 3f, humic acid-like organics in the raw water contributed the highest fluorescence intensity. Two different coagulants exhibited similar removal trends for fluorescent components, where the rate of fluorescence intensity decay decreased with increasing doses. Based on the information shown in Table S2, the proportion of humic substances decreased after coagulation, while the proportion of protein-like organics increased. It is evident that coagulation for DOM removal is a highly selective process, with higher effective removal of humic substances due to the strong interaction between phenolic and carboxyl functional groups in humic substances and metal hydroxides [27]. When the dose of both coagulants was set at 5 mg·L^−1^, it was observed that FeCl_3_ exhibited greater removal efficiency for total fluorescence intensity compared to PAC. Due to aluminum’s higher solubility in seawater compared with iron, lower doses result in reduced floc formation, rendering PAC less effective in removing fluorescent DOM under these circumstances [28]. Nevertheless, as the coagulant dose increased, the variation in fluorescence intensity became less pronounced. Notably, the EEM spectra at a PAC dose of 30 mg·L^−1^ (Figure 3c) bore resemblance to those at a FeCl_3_ dose of 10 mg·L^−1^ (Figure 3e). This is potentially because excessive coagulant may fail to enhance the removal efficiency of select fluorescent organic compounds that demonstrate resistance to conventional coagulation processes [29].

**Figure 4:**
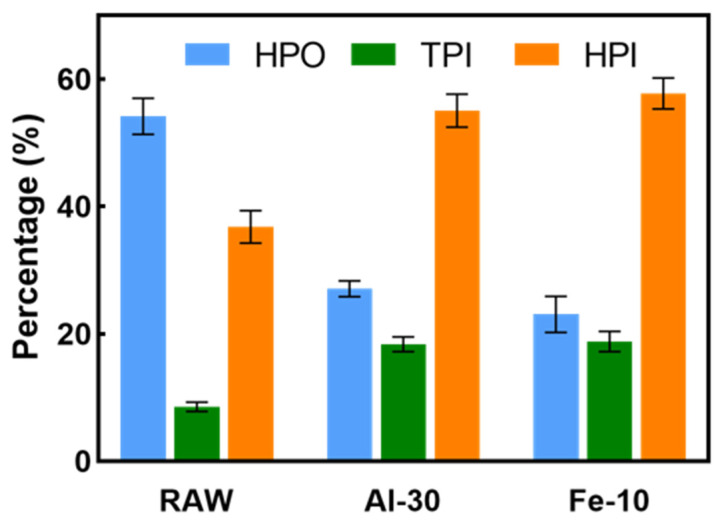
The content of hydrophilic, transphilic and hydrophobic fractions of organics in raw water and water treated with 30 mg·L^−1^ PAC and 10 mg·L^−1^ FeCl_3_.

Figure 4 depicts the content of hydrophilic, transphilic and hydrophobic fractions of DOM in unpretreated water samples, as well as those in water pretreated with 30 mg·L^−1^ PAC (referred to as Al-30) and 10 mg·L^−1^ FeCl_3_ (referred to as Fe-10). The removal rate of the HPO fraction was higher than that of the HPI fraction in both coagulants. For PAC, the removal rates were 82.4% for the HPO fraction and 47.5% for the HPI fraction, while for FeCl_3_, they were 85.5% for the HPO fraction and 45.5% for the HPI fraction. This differential removal efficiency is attributed to the higher charge density of the HPO fraction compared to the HPI fraction, leading to enhanced interactions between HPO and polyvalent metal ions, thereby increasing the effectiveness of traditional coagulation pretreatment [30,31].

Given that the efficiencies of the two coagulants were comparable, we selected 10 mg·L^−1^ FeCl_3_ as the coagulant for the following study, due to its low dose.

### 3.2. Removal Performance in the C-S and C-U Pretreatment

**Figure 5:**
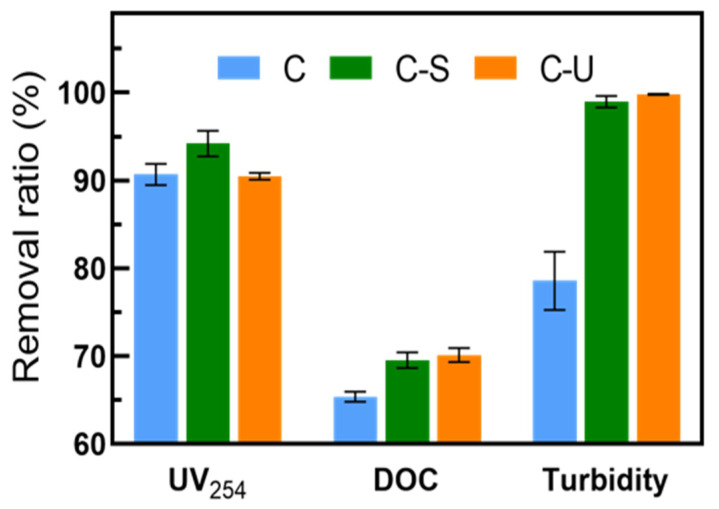
Removal ratio of UV_254_, DOC, and turbidity in the coagulation (10 mg·L^−1^ FeCl_3_), C-S and C-U pretreatments.

Figure 5 displays the removal ratios for turbidity, UV_254_ and DOC. Both C-S and C-U pretreatments exhibited great performance in removing particles, colloids, and suspended substances, with a turbidity removal rate of up to 99%, demonstrating excellent retention of inorganic particles. C-U pretreatment showed reduced efficiency in eliminating aromatic organic substances, notably in UV_254_ absorption, compared to C-S pretreatment. However, C-U pretreatment achieved a slightly higher DOC removal ratio compared to C-S pretreatment.

Considering comprehensive water quality indicators, both C-U and C-S pretreatments contributed to the removal of pollutants. Disregarding the impact of UV-absorbing compounds and focusing solely on DOM removal, C-U pretreatment performed better than C-S pretreatment. Lower SDI values and reduced error fluctuations also indicated a greater stability of C-U pretreatment with a higher quality of effluent.

**Figure 6:**
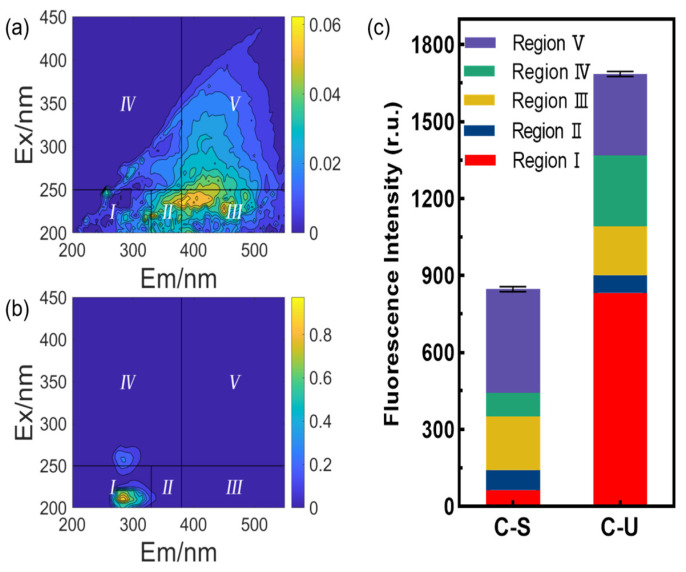
EEM spectra and FRI analysis of organics in effluent after treatment with (**a**) C-S or (**b**) C-U pretreatment; (**c**) fluorescence intensity of effluent after treatment with C-S or C-U pretreatment (Regions I, II, III, IV, and V were noted in the EEM spectras, corresponding to tyrosine protein-like organics, tryptophan protein-like organics, fulvic acid-like organics, soluble microbial by-product-like (SMP-like) organics, and humic acid-like organics, respectively).

The analysis of the distribution of fluorescent components in the effluent from C-S and C-U pretreatments (Figure 6) revealed that each pretreatment had diverse effects on the removal of organic substances from different fluorescent regions. C-S pretreatment can remove SMP-like organics more significantly than C-U pretreatment, while C-U pretreatment is more effective in removing large molecular weight humic acids. Notably, the proportion of tyrosine protein-like organics in the C-U pretreatment effluent significantly increased, showing a low retention of protein-like organics. This phenomenon may result from a combination of three factors. First, the retention of organic substances by UF membranes relies not only on their pore size but also on their interaction with organic substances. Research indicates that proteins have a stronger tendency to adsorb in hydrophobic environments [32]. Consequently, the pronounced hydrophilicity of UF membranes made of PES may lead to a relative lack of protein adsorption efficiency. Second, under conditions of high ionic concentration, the compression of the electrical double layer (EDL) effect can reduce the hydrodynamic radius of proteins such as BSA, thereby decreasing the protein retention rate of UF membranes [33]. In addition, an increase in ionic concentration may accelerate the solubility of proteins, thereby indirectly leading to a decrease in the protein removal rate in the UF process [34]. Overall, C-S pretreatment exhibited a more pronounced overall removal effect on fluorescent DOM, a conclusion that aligns with the organic matter removal trend evaluated through UV_254_ absorption values.

After the operation, the specific surface area of the quartz sand filter medium increased from 0.076 m^2^·g^−1^ to 0.086 m^2^·g^−1^, indicating changes in its surface characteristics. SEM and EDX analyses (Figure S2) revealed that the original quartz sand surface was relatively smooth and was primarily composed of silicon and oxygen. After filtration, certain areas of the quartz sand surface were covered with a thin layer of flocculent material, leading to the emergence of C, N, and Fe. Data from Table S1 revealed that the concentration of dissolved iron in the effluent from SF was lower compared to that in the influent. Given the influent’s pH of approximately 5.5, part of the dissolved iron likely precipitated as iron oxides onto the quartz sand surface. These iron oxides are capable of adsorbing DOM through mechanisms such as ligand exchange and hydrophobic interaction [35]. Their deposition not only increases the specific surface area of the quartz sand but also expands the area available for adsorption. Consequently, the removal of DOM by SF was the result of a combined effect of physical interception and chemical adsorption.

**Figure 7:**
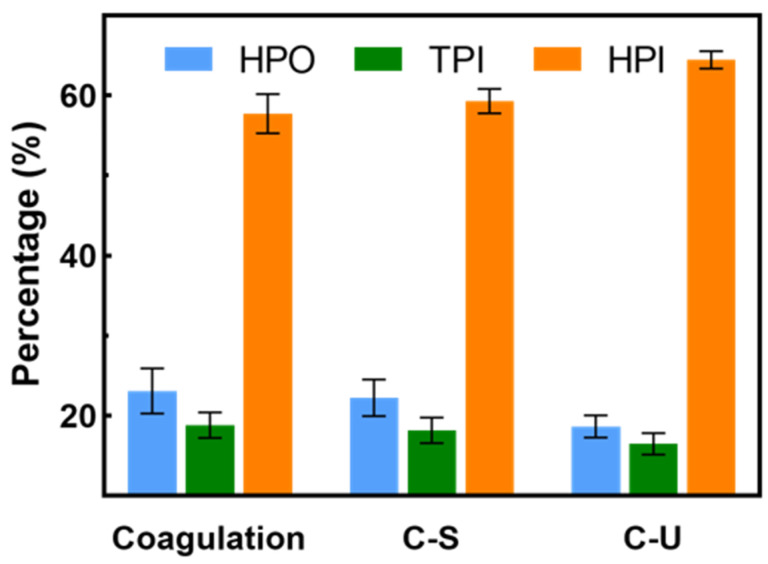
The content of hydrophilic, transphilic and hydrophobic fractions of organics in the effluent pretreated with coagulation (10 mg·L^−1^ FeCl_3_), C-S or C-U pretreatment.

Figure 7 shows the content of hydrophilic, transphilic and hydrophobic fractions of DOM in the effluent of C-U and C-S pretreatments. After C-S or C-U pretreatment, the percentages of HPO and TPI fractions slightly decreased, while the percentage of the HPI fraction increased, indicating that both pretreatments could reject some hydrophobic and transphilic DOM but had limitations in removing the HPI fraction. Notably, the proportion of HPI increased in the C-U effluent. As shown in Figure S3, both the C-S and C-U pretreatments effectively removed large particles unsettled during the coagulation stage. The particle sizes of the remaining organic substances further decreased after C-U pretreatment.

### 3.3. Effect of Integrated Pretreatment on RO Fouling

**Figure 8:**
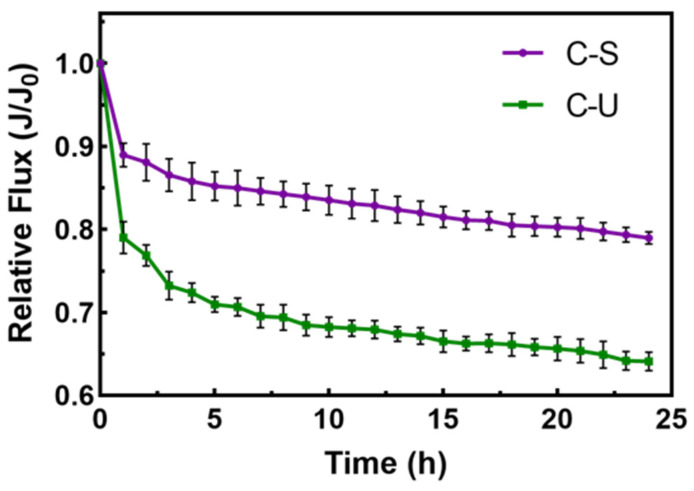
Effect of integrated pretreatments on normalized RO membrane flux.

The effluent from C-S or C-U pretreatment was used as the influent for the RO system. The effect of pretreatment technologies on RO membrane fouling was investigated by analyzing changes in the performance of RO membranes. In Figure 8, the decline in the RO membrane flux after C-S pretreatment was relatively gradual, with a decrease of approximately 20% after 24 h. In contrast, after C-U pretreatment, the RO membrane flux was only 65% of the initial flux at the end of 24 h, indicating that effluent from C-U pretreatment resulted in more-severe RO membrane fouling.

### 3.4. RO Membrane Fouling Analysis

**Figure 9:**
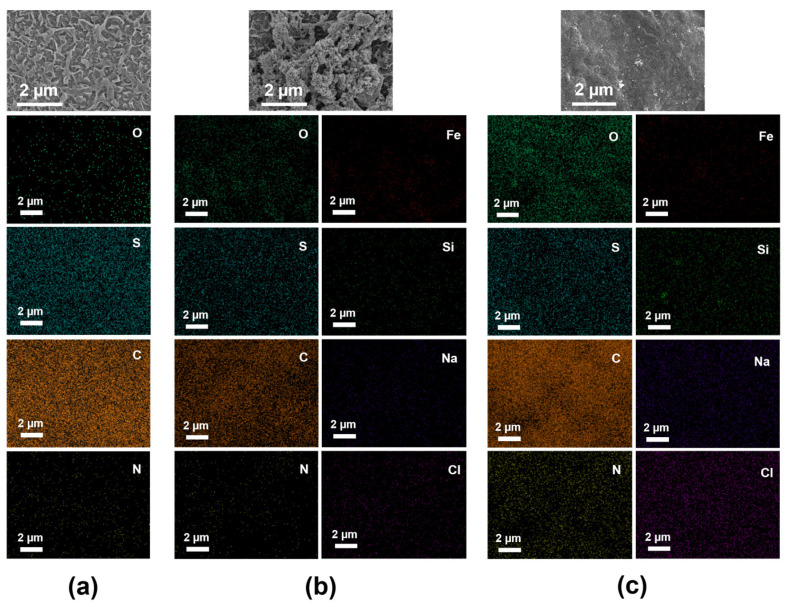
SEM images and their corresponding EDX mapping images. (**a**) Virgin RO membrane, (**b**) fouled RO membrane after C-S pretreatment, and (**c**) fouled RO membrane after C-U pretreatment.

SEM images and EDX analysis (Figure 9) can reveal the surface changes in the microstructure and chemical composition of the RO membrane. It was found that the surface of virgin RO membrane was rough and orderly (Figure 9a). After operation with the feed pretreated by C-S pretreatment, the RO membrane surface developed a loose and porous layer of organic pollutants, as illustrated in Figure 9b. Conversely, after operation with the feed pretreated by C-U pretreatment, pollutants on the RO membrane were more tightly packed, as illustrated in Figure 9c. Organic substances capable of penetrating the UF membrane tend to accumulate in the valleys of the RO membrane, leading to a denser fouling layer on the RO membrane surface [36]. After operation with the feed pretreated by C-U pretreatment, the N element proportion in the dense cake layer on the RO membrane surface rose by approximately 13% compared to the original membrane (Figure S4).

Table 1 shows the changes in the hydrophobicity, surface charge and roughness of virgin RO membranes and fouled RO membranes with pretreated seawater. The results for the water contact angle showed the following order: RO fouled with feed pretreated by C-U (29.4 ± 1.4°) < virgin RO (35.2 ± 1.3°) < RO fouled with feed pretreated by C-S (52.3 ± 2.5°). A higher water contact angle represents higher hydrophobicity of the membrane surface [37]. An increase in the water contact angle of RO membrane fouled with feed pretreated by C-S suggested that C-S pretreatment led to prevention of absorption of hydrophilic fractions (present in the feed water) on the membrane surface. Table 1 also presents the variation in zeta potential of virgin RO membranes and fouled RO membranes. The zeta potential of RO membrane fouled with pretreated feed increased after pollutant deposition, suggesting that the ionizable acidic groups associated with the membrane might be covered by DOM components that were less charged or uncharged. Moreover, the zeta potential of the RO membrane fouled with feed pretreated by C-U significantly increased, indicating the deposition of more neutral organic substances on its surface. Neutral organic substances can easily deposit on the negatively charged RO membrane surface [38]. Such deposition diminishes electrostatic repulsion between hydrophobic fractions and RO membrane, thereby encouraging organic accumulation and intensifying membrane fouling. The roughness of RO membrane fouled with feed pretreated by C-S increased, whereas it slightly decreased after C-U pretreatment, corresponding to a denser fouling layer on its surface, as depicted in Figure 9c.

## 4. Discussion

The organic pollutants on the RO membrane surface fouled with feed pretreated by C-S were relatively loose. However, the fouling layer became more compact after C-U pretreatment. The effluent from C-U pretreatment contained a higher proportion of tryptophan protein-like organics, SMP-like organics and colloidal DOM than that from C-S pretreatment. Tryptophan protein-like organics exhibit the strongest interaction with RO membrane compared to other fluorescent components [39]. SMP-like organics are considered to have strong fouling potential, and their fluorescence intensity showed a good positive correlation with the degree of fouling on SWRO membranes [40]. The deposition of these organics forms a dense cake layer on RO membrane, leading to a significant flux decline and irreversible fouling.

In summary, we list the various indicators in Table S2. Both C-S and C-U pretreatments effectively removed turbidity. Among them, C-S performed better in removing aromatic compounds, while C-U was slightly ahead in removing DOC, and the SDI value of the effluent from the C-U pretreatment was lower.

However, notable differences existed between the two integrated pretreatment technologies in the removal of fluorescent components. The effluent from the C-S pretreatment contained the lowest proportions of tyrosine protein-like organics and SMP-like organics, whereas after C-U pretreatment, the proportions of such components were the highest.

## 5. Conclusions

This study investigated the effectiveness of two integrated pretreatment technologies, C-S pretreatment and C-U pretreatment, in removing DOM from seawater, as well as their impacts on RO membrane performance. RO experiments showed that RO had more severe membrane fouling and higher flux decline with feed water pretreated by C-U than with that pretreated by C-S. Specifically, C-U resulted in a compact fouling layer, contrasting with the porous layer formed after C-S pretreatment. Coagulation was found to selectively target DOM, particularly excelling in the removal of the hydrophobic fractions and humic acid-like substances. Both integrated pretreatment technologies significantly improved RO feed water quality according to comprehensive indices; nonetheless, they exhibited distinct patterns in DOM component removal, which directly influenced the fouling propensity of the effluent and, consequently, RO performance. C-S pretreatment effectively removes compounds associated with UV_254_, whereas C-U pretreatment significantly increases the levels of tyrosine protein-like organics, SMP-like organics, and small-sized organics in the effluent. Their deposition on the RO membrane surface forms a dense cake layer. In conclusion, careful consideration should be given to selecting suitable water quality testing and pretreatment technologies, as well as their integration, based on the specific characteristics of the water source. When the feed water contains a substantial proportion of protein-like organics or SMP-like organics, SF can be considered as a viable pretreatment option for SWRO. Conversely, if the feed water is rich in humic substances, UF may prove more efficacious in delivering higher-quality feedwater for SWRO processes.

## Figures and Tables

**Table 1:** Changes in the hydrophobicity, surface charge and roughness of virgin RO membranes and RO membranes fouled with pretreated seawater.

	Virgin RO Membrane	RO Membrane after C-S Pretreatment	RO Membrane after C-U Pretreatment
Water contact angle (°)	35.2 ± 1.3	52.3 ± 2.5	29.4 ± 1.4
Zeta potential at pH 5.5 (mV)	−20.8 ± 0.7	−17.3 ± 1.4	−9.9 ± 1.3
Surface roughness (nm)	50.7 ± 3.1	64.3 ± 4.2	42.4 ± 3.6

## Data Availability

The original contributions presented in the study are included in the article and Supplementary Materials, further inquiries can be directed to the corresponding authors.

## Supplementary Materials

The following supporting information can be downloaded at https://www.mdpi.com/article/10.3390/membranes14060125/s1, Figure S1: Static images of the contact angle for the UF membrane; Figure S2: SEM images and chemical precipitations by EDX. SEM images of (a) clean quartz sand and (b) SEM images of fouled quartz sand; EDX analysis of (c) clean quartz sand and (d) EDX analysis of fouled quartz sand; Figure S3: Size distribution of small particles in the raw water and effluent pretreated with Coagulation, C-S or C-U pretreatment; Figure S4: EDX anlysis of (a) virgin RO membrane, (b) fouled RO membrane after treatment by C-S pretreatment, and (c) fouled RO membrane after treatment by C-U pretreatment; Table S1: Characteristics of the raw water and effluent pretreated with coagulation (10 mg·L^−1^ FeCl_3_), C-S and C-U pretreatments; Table S2: Changes in the water characteristics of raw seawater, coagulation (10 mg·L^−1^ FeCl_3_) supernate, C-S pretreatment and C-U pretreatment.
