# Supplementary material for: Comparison of Coagulation-Integrated Sand Filtration and Ultrafiltration for Seawater Reverse Osmosis Pretreatment
Source: Membranes (Basel). 2024 May 29;14(6):125. doi: 10.3390/membranes14060125 (PMC11205350; doi:10.3390/membranes14060125)
Supplement: Supplementary file 1 [file membranes-14-00125-s001.zip › membranes-3012641-supplementary.pdf]

membranes

Supplementary Materials

Comparison of Coagulation-integrated Sand Filtration and Ultrafiltration for Seawater Reverse Osmosis Pretreatment

Qingao Li, Lixin Xie *, Shichang Xu and Wen Zhang *

Tianjin Key Laboratory of Membrane Science and Desalination Technology, State Key Laboratory of Chemical Engineering , School of Chemical Engineering and Technology, Tianjin University, Tianjin 300350, China; lqa@tju.edu.cn (Q.L.); xu_sc1@tju.edu.cn (S.X.)
* Correspondence: xie_lixin@tju.edu.cn (L.X.); zhang_wen@tju.edu.cn (W.Z.)

S.1

To ascertain the contact angle of the UF membrane, a segment was first dried at 40°C for 24 hours. It was then delicately sliced at its midpoint using a sharp blade, and affixed onto a microscope slide with double-sided tape, and the contact angle of the UF membrane was subsequently measured utilizing the sessile drop method. Measurements were repeated 3 times to calculate the average contact angle. Figure 3 presents static photographs captured during the tests, with their respective contact angles being 45.1° (Figure S1a), 41.6° (Figure S1b), and 40.8° (Figure S2c). Consequently, the mean contact angle for the UF membrane sourced from the manufacturer is determined to be 42.5°.

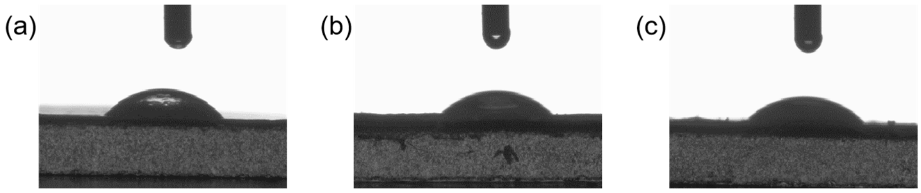

Figure S1. Static images of the contact angle for the UF membrane.

S.2

As can be seen from Table S1, it is evident that the water samples after coagulation has a turbidity of 3.2 NTU, and an SDI of 4.4, failing to meet the inlet requirements of RO. Hence, the supernate must undergo subsequent pretreatment. The SDI values are significantly reduced to below 3.5 after C-S pretreatment and below 2 after C-U pretreatment.

Table S1. Characteristics of the raw water and effluent pretreated with coagulation (10 mg•L^{-1} $FeCl_3$), C-S and C-U pretreatments.

	Raw Water	Coagulation	C-S	C-U
Turbidity (NTU)	15.0 ± 0.5	3.2 ± 0.6	0.2 ± 0.05	0.03 ± 0.01
Zeta potential (mV)	-13.5 ± 1.2	-4.6 ± 0.4	-5.2 ± 0.9	-3.2 ± 0.6
SUVA	4.8 ± 0.5	1.3 ± 0.3	0.94 ± 0.3	1.5 ± 0.2
Dissolved iron (μg•L^{-1})	N.D.	170.1 ± 0.5	111.75 ± 0.4	133.6 ± 0.2
SDI		4.4 ± 0.6	3.2 ± 0.3	1.8 ± 0.2

Table S2. Changes in the water characteristics of raw seawater, coagulation (10 mg•L^{-1} $FeCl_3$) supernate, C-S pretreatment and C-U pretreatment.

		Raw seawater	Coagulation supernate	C-S pretreatment effluent	C-U pretreatment effluent
Comprehensive water quality indices	UV_{254} (abs)	0.185	0.017	0.011	0.018
	DOC (mg•L^{-1})	3.82	1.33	1.16	1.14
	Turbidity (NTU)	14.7	3.2	0.05	0.03
	SDI		4.4	3.24	1.86
Fluorescence response percentage	Tyrosine protein-like organics	0.075	0.089	0.13	0.75
	Tryptophan protein-like organics	0.21	0.28	0.34	0.076
	Fulvic acid-like organics	0.17	0.19	0.27	0.060
	Soluble microbial by-product-like organics	0.19	0.25	0.12	0.086
	Humic acid-like organics	0.35	0.19	0.14	0.028

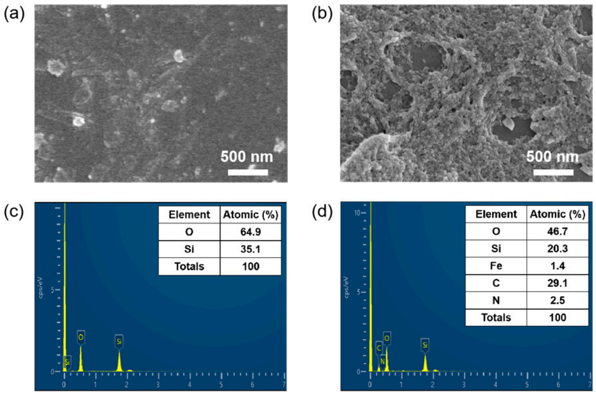

Figure S2. SEM images and chemical precipitations by EDX. SEM images of (a) clean quartz sand and (b) SEM images of fouled quartz sand; EDX analysis of (c) clean quartz sand and (d) EDX analysis of fouled quartz sand.

The particle size distribution was utilized to characterize the particulates in the raw seawater and the effluent from various pretreatment processes, as shown in Figure S3. Both the C-S and C-U ptetreatments effectively removed large particles unsettled during the coagulation stage. The particle size of the remaining organic substances further decreased after C-U pretreatment.

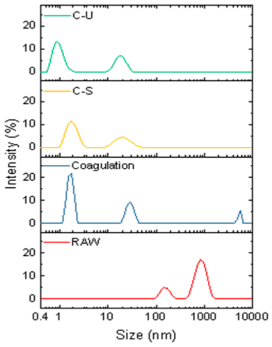

Figure S3. Size distribution of small particles in the raw water and effluent pretreated with Coagulation, C-S or C-U pretreatment.

S.3

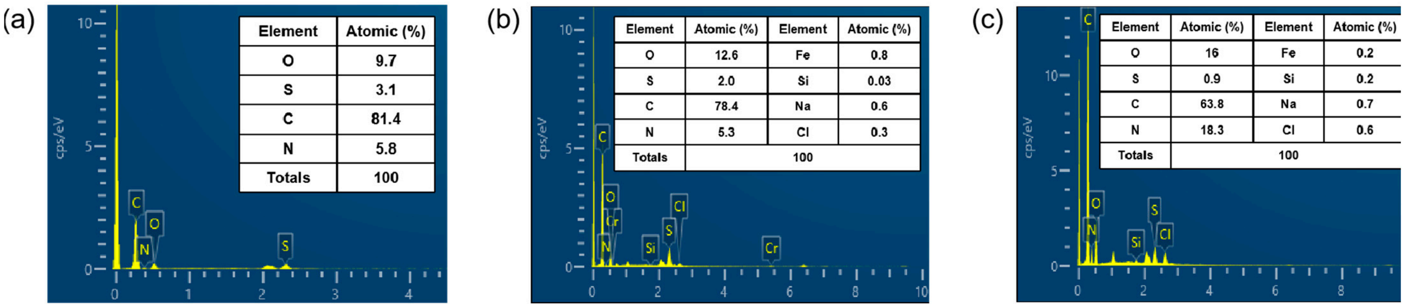

Figure S4. EDX anlysis of (a) virgin RO membrane, (b) fouled RO membrane after treatment by C-S pretreatment, and (c) fouled RO membrane after treatment by C-U pretreatment.
